# Exploring Junior Residents’ Barriers in Mobilizing Social Capital and Their Coping: A Qualitative Ego Social Network Study

**DOI:** 10.5334/pme.1629

**Published:** 2025-07-30

**Authors:** Gerbrich Galema, Götz J. K. G. Wietasch, Debbie A. D. C. Jaarsma, Jasperina Brouwer

**Affiliations:** 1 University of Groningen, University Medical Center Groningen (UMCG), and part of Lifelong Learning, Education and Assessment Research Network (LEARN), She is an anesthesiologist at the department of anesthesiology, NL; 2 University of Groningen, UMCG, Department of Anesthesiology, He is also part of LEARN, NL; 3 Utrecht University, Faculty of Veterinary Medicine, She is also part of LEARN, NL; 4 University of Groningen, Faculty of Behavioral and Social Sciences, Department of Educational Sciences, with expertise in qualitative and quantitative research and social network analysis, NL

## Abstract

**Background:**

Residents rely on social support from team members to navigate patient care, while also facing challenges integrating into the healthcare team. Although prior research has explored help-seeking behavior, little attention has been given to how residents overcome barriers to mobilizing social resources. This study explores how, in daily clinical practice, do residents perceive and respond to barriers in accessing and making use of social capital during challenging situations?

**Methods:**

We used a mixed-methods social network approach. Twenty-nine junior residents from various specialties participated in qualitative interviews, supplemented by ego (personal) networks to encourage respondents’ reflection on social relationship dynamics.

**Results:**

Qualitative ego network analysis revealed that residents encounter significant physical and psychological barriers, such as uncertainty and the perceived costs of seeking support, which limit access to crucial information and expertise. Quantitative ego network analysis showed that residents predominantly turned to supervisors, nurses, and peers in challenging situations. However, interviews highlighted the complexity of supervisory relationships, shaped by concerns about career impact. To navigate these barriers emotional support was sought from friends, parents, and close colleagues, while expertise was sought from supervisors, senior residents, and nurses.

**Conclusion:**

Residents face significant barriers in mobilizing social capital, particularly with supervisors. To cope, they draw on different parts of their network for emotional support and expertise. Our results suggest that existing help-seeking models, such as Borgatti and Cross’s, require refinement: power dynamics contribute to the perceived ‘costs’ of help-seeking, and uncertainty about others’ supportive potential reflects the influence of role ambiguity and relational comfort.

## 1. Introduction

Medical residents experience their residency as a period of intense professional growth, but also as a period marked by high workloads, complex decision-making, and emotional challenges, factors that can contribute to exhaustion and burnout [1]. To navigate these demands, residents must effectively seek help from their team members. While supervisors are traditionally the primary source of educational and training support [2], support from other team members, such as peers and nurses, is equally essential for the residents’ learning and well-being [3,4]. The support of other team members can involve providing valuable learning opportunities, enabling residents to deepen their understanding of specific pathologies [5], acquiring technical skills [6], and integrating into the healthcare team [3,5,7]. However, becoming part of the healthcare team requires navigating the hidden curriculum, i.e., the unwritten rules and professional norms specific to each department and specialty [8,9]. These unwritten rules, such as the belief that residents must be independent decision-makers, can discourage help-seeking. As a result, residents may feel uncertain about when, how, and whom to approach for support. In the hierarchical healthcare system, residents may hesitate to seek help, knowing that supervisors also evaluate their performance [3,10,11]. Additionally, frequent rotations to new environments hinder relationship-building with supervisors [12,13,14]. Residents are more likely to seek help if (1) supervisors are not occupied; (2) residents work physically close to peers, nurses, and supervisors; and (3) residents perceive their colleagues as approachable [3,11]. While residents and nurses are often perceived as more approachable than supervisors, a supervisor’s approachability depends largely on previous interactions [3,11]. Thus, despite the importance of social support, residents face barriers in help-seeking, particularly within hierarchical medical settings. Research has explored these barriers in clinical decision-making and supervision [3,11], but the mechanisms by which residents actively mobilize their networks to overcome these barriers remain unclear, specifically how residents select team members when they need support. Understanding how residents mobilize their networks is critical for shaping educational environments that empower residents to seek support, thrive under pressure, and provide safe patient care. Existing models of information or advice-seeking can help explain the factors influencing whom individuals turn to for support.

Information and advice-seeking behavior was previously conceptualized by Borgatti and Cross in organizational settings. Their model emphasizes four key factors: knowing (what others know), valuing (the relevance of that knowledge), access (how easily they can be reached), and cost (perceived effort or risk). They showed that knowing, valuing, and access significantly predicted information and advice-seeking behavior, while cost did not, suggesting that relational dynamics, like trust and access, matter more than perceived costs [15]. In medical residency, where hierarchical structures and frequent rotations shape professional interactions, this model is useful to examine how residents seek information, advice, and support. Residents navigate a complex social environment with access to various actors in their networks, including peers, nurses, supervisors, and personal contacts such as family and friends [4,10,16]. While research has explored the barriers residents face in seeking clinical support [3,11], little research has addressed how they actively mobilize their networks to overcome these barriers. Moreover, although Borgatti and Cross’s research has primarily been applied to information and advice-seeking, its relevance to broader help-seeking behavior, particularly within the unique social and professional constraints of medical training, remains underexplored [3,11].

In the current study, we use a social network and social capital perspective to explore how residents perceive and respond to barriers in accessing and making use of social capital during challenging situations [17,18,19]. A social network, in this context, refers to the relationships (or ties) that residents form during their training [19], including both informal (e.g., friends, peers) and formal connections (e.g., supervisors, nurses, peer residents). These networks create the structure through which social capital flows [19,20]. Social capital refers to the resources, such as expertise, information, and support, that medical residents can access through their social networks [18,21]. Within residency training, social capital can help residents navigate a complex clinical environment, acquiring technical skills or becoming part of a team, which they might not be able to achieve otherwise [18,21]. However, these resources are not equally accessible to all residents; rather, they depend on the quality, structure, and positioning of their relationships within the network. Social capital theory thus provides a useful interpretive lens, helping us understand why some residents are better able to cope with challenges than others, based on the configuration of their social ties [19,20]. While prior research has highlighted the importance of supervision [2,10], communities of practice [5], and personal relationships [16], fewer studies have explicitly examined how residents’ networks function as sources of social capital or how barriers to accessing these networks affect learning and well-being. To better understand this, we conducted a qualitative ego network analysis approach, which focuses on the connections of an individual (the “ego”), residents’ perceptions, and their personal networks [22]. In ego networks, the resident is positioned at the center and identifies other actors who possess resources that can be mobilized for various purposes, such as progressing through training or becoming familiar with the department’s unwritten rules, thereby facilitating access to social capital [22]. This approach allows us to explore not only who residents rely on but also how and why certain relationships become valuable in overcoming barriers in the workplace. Our main research question is:

In daily clinical practice, how do residents perceive and respond to barriers in accessing and making use of social capital during challenging situations?

## 2. Methods

Our research reflects an interpretivist paradigm, recognizing that reality is subjective and context-specific and that there is no ultimate truth [23].

### 2.1. Design

**Figure 1:**
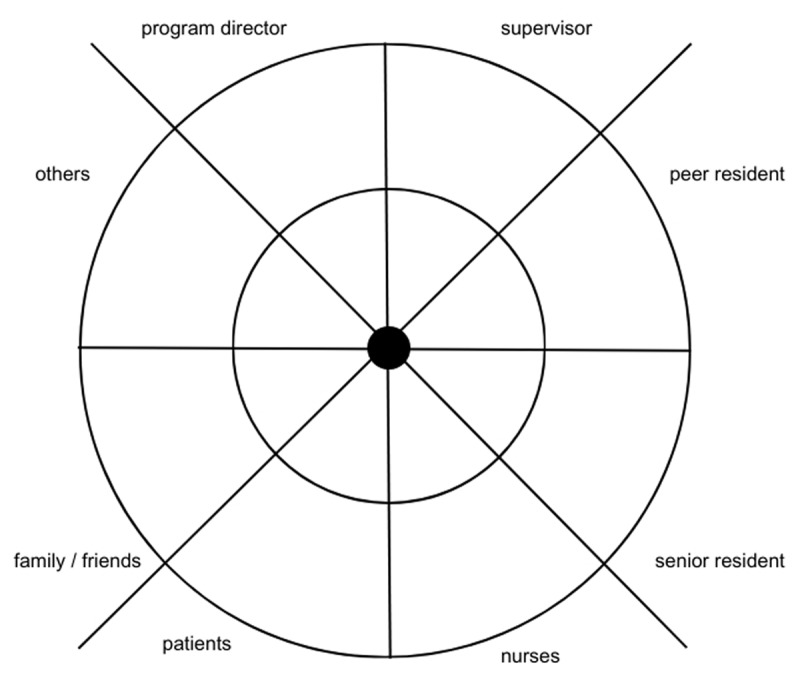
Empty sociogram.

To address the research question, we used a mixed-methods research design, comprised of an assorted analysis [24]. This analysis is characterized by a secondary analysis of 16 qualitative interviews, which informed the subsequent data collection of 13 qualitative interviews [24]. The secondary analysis enabled us to identify residents’ social capital and probed the barriers residents faced in accessing and utilizing this social capital in daily practice. The analysis showed, however, that we required more information power from the data to identify how residents perceive and respond to barriers in accessing and making use of social capital during challenging situations [25]. In the subsequent interviews, we asked for residents’ reactions to two challenging situations: (1) instances in which they encountered challenges in accessing and making use of social capital (negative situations) and (2) instances in which they easily accessed and made use of social capital (positive situations). We supplemented these interviews with ego network sociograms [26] to visualize and analyze the structure of each resident’s social network. An ego social network analysis (SNA) enhances the understanding of how relationships shape access to resources [20,22,27,28,29]. In this study, we employed a concentric circle network visualization (Appendix 1, Figure 1; adapted from [30,31]) to construct ego network sociograms, allowing participants to map their social connections based on perceived closeness and role within their network. This approach enabled us to gain deeper insights into how the social environment influences individual residents.

### 2.2. Sample

We selected junior residents from various specialties and hospitals in the Netherlands using convenience and purposive sampling. The sample consisted of residents not in training, who have completed medical school but do not yet have a postgraduate specialty training position, and second year specialty training residents, who have begun their specialty training [32]. We purposefully sampled typical cases of residents not in training because of their unique position within the social system of physicians, as explained in the next section.

### 2.3. Setting

After graduation from medical school, residents work for approximately 3.5 years as “residents not in training” before starting their specialty training [33]. This period allows them to explore potential specialties, gain experience, and apply for specialty training. Unlike formal training, this phase lacks a structured curriculum, formal learning goals, or an official program director [32,34]. Faculty use this period to assess the resident’s suitability for specialty training. Although performance-based criteria are key in postgraduate selection, implicit social processes like group fit and selection committees’ personal beliefs may play a role [35]. Specialty training lasting three to six years, includes a formal curriculum, individualized training plan, regular feedback, and (programmatic) assessment through EPAs [34,36]. The curriculum comprises various 3- to 12-month rotations that are contextualized in academic and nonacademic teaching hospitals [36].

### 2.4. Ethical Approval

The study was ethically approved by the Central Ethics Review Board of the University Medical Center Groningen (register number: 20190064). All participants provided informed consent.

### 2.5. Data Collection

Interviews were recorded, pseudonymized, and transcribed verbatim. Participants reviewed their transcripts but made no corrections. Two different interview guides were used to capture detailed insights into residents’ experiences mobilizing social capital. Interview guide 1 (Appendix 2) focused on residents’ adaptation strategies during their first year and the support they received [37]. Interview guide 2 (Appendix 3) focused on scenarios where residents either struggled or succeeded in mobilizing social capital, which involves instances of help-seeking. In this context, the scenarios can include both information- and advice-seeking behaviors. While help-seeking can be a broader concept, we emphasize that residents either seek information (e.g., clinical knowledge), or advice (e.g., validation of their decisions), both of which are encompassed by Borgatti and Cross. The interviews were enriched by using sociograms.

The interviewer (first author) asked the residents to describe a situation. Participants then constructed a visual map of their personal network, known as an ego network sociogram, using adhesive notes. This social networks mapping started with a name generator [29]: in which participants identified key individuals (known as alters) in their personal network. An *alter* refers to any person with whom the ego (i.e. the resident) has a meaningful social relationship [26], such as colleagues, supervisors, or friends. Participants placed themselves at the center of the diagram and arranged alters around them, positioning closer connections nearer the center and more distant or less accessible connections farther away.

We then explored the nature of each relationship, the resources exchanged, strategies to mobilize social capital, and the connections between alters (referred to as inter-alter ties) [28,29]. These inter-alter connections provide insight into underlying relational dynamics that shape access to support [28]. In addition to name generating and interpreting, the inter-alter questions are essential for understanding the social network structure, specifically how the ego (i.e., the resident) is embedded within the network and which alters could be mobilized [22,29]. Although participants could not modify the sociograms during the interview, they were allowed to create separate diagrams for negative and positive situations. Comparing these maps helped participants reflect on why they turned to certain actors in one situation but not in another, revealing potential gaps in their networks.

### 2.6. Data Analysis

Four researchers (GG (all), AN (16), TA (3) and WL (4)) independently coded the interviews and sociograms, discussing discrepancies until consensus was reached. Codes were refined to capture barriers in mobilizing social capital, strategies to overcome these barriers, and sociogram distances. Based on the information power criterion, the 29 interviews were deemed sufficient to answer the research question [25,38]. To deepen our analysis, we applied Borgatti and Cross’s model of information and advice-seeking, which considers factors as knowledge, valuing that knowledge, timely access, and the perceived cost of information and advice-seeking [15]. Atlas.ti software was used for data management and analysis [39], and the final themes were discussed within the research team to ensure a thorough understanding of residents’ social capital dynamics. GG drafted the results section, which was further refined through team discussions.

The sociograms were analyzed in terms of social network descriptives [29]. We assessed the sociograms by describing the network size (alter count), the range of network sizes across participants, and the distribution of alters across the concentric circles. We also examined the types of alters most frequently identified, distinguishing between the different role-groups (supervisors, peer residents, senior residents, nurses, patients, family/friends, program directors, others,). Furthermore, we explored who was most often positioned in the closest, middle, and outer circles, providing insight into the perceived accessibility of different sources of support. The quantitative information was used to increase the breadth of the data and support the trustworthiness of the qualitative information [28].

### 2.7. Reflexivity and Research Team

The first author (GG), a PhD in medical education and a resident, is familiar with the research context of the secondary analysis [40]. She interviewed participants, leveraging her shared experiences for a deeper understanding of the setting. To challenge her assumptions, GG discussed her findings with JB, who was not influenced by previous resident experience. The second author (GW), a former anesthesiology program director, provided a supervisor’s perspective, while the last author (JB), an assistant professor in educational sciences with social network analysis expertise, added a social science perspective. JB’s experience as a former nurse who worked with residents also enriched the analysis. The team’s diverse clinical backgrounds and reflective practices during analysis helped uncover new perspectives on the discussions and revealed overlooked aspects of the data, enhancing the authenticity of their findings [41,42]. The team was complemented by an experienced qualitative researcher and professor in (veterinary) medical education (DJ) and medical students (AN, WL, TA).

## 3. Results

For this study, we recruited 29 residents through email, LinkedIn, and personal networks. Interviews lasted 56 to 78 minutes (average of 68 minutes). Table 1 details the characteristics of the entire sample.

**Table 1:** Characteristics of interviewees *(n = 29)*.


PARTICIPANT CHARACTERISTICS	TOTAL (n)

**Hospital**	

Academic	18

Non-academic teaching	11

**Specialty**	

Surgical	9

Non-surgical	20

**Months of clinical experience**	

Residents not in training	13

Specialty training residents	16

**Gender**	

Female	19

Male	10


**Figure 3:**
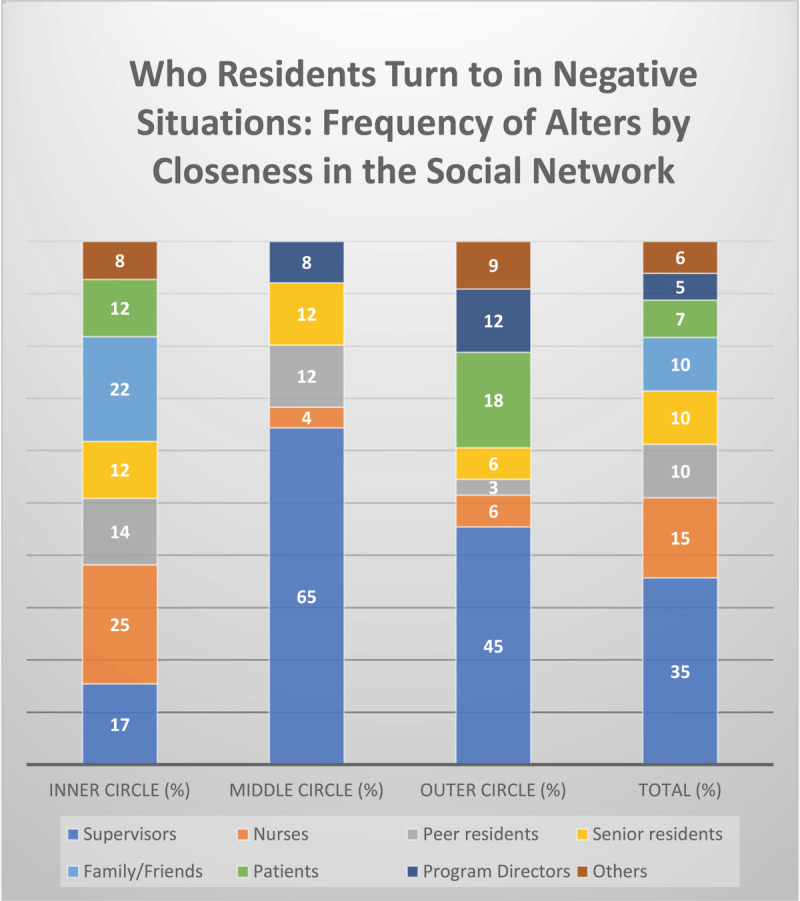
Visual representation of Residents’ Social Capital Mobilization in Negative Situations. This figure presents an overview of residents’ social capital mobilization networks in negative situations. It shows which groups of alters residents placed in the inner, middle, and outer circles. The final bar displays the overall distribution of these groups across all circles.

Our findings reveal how residents perceive and respond to barriers in accessing and making use of social capital during challenging situations. Residents encountered a range of physical and psychological barriers that impeded their ability to manage complex patient cases, challenging learning environments, career goals, and professional interactions. In the following sections, we discuss the barriers through the lens of Borgatti and Cross’s information and advice-seeking model, which includes challenges such as not-knowing one another, (un)awareness of the supportive potential, difficulty accessing the right person, and the perceived high costs of seeking support. The findings are supported by respondent quotes and sociograms, illustrating the complexities of navigating challenging situations. Figures 3, 4, and Table 2 (Appendix 4) provide an overview of the two competing scenarios and outline the concentric circle data collection method. The sociograms contrast the dynamics of social capital mobilization in negative versus positive situations, offering a structured comparison of the alters identified by residents in each context. We refer to the Figures and Table 2 throughout to support our claims.

### 3.1 (Not) Knowing One Another

Residents encountered physical barriers when seeking help from unfamiliar healthcare professionals. The lack of daily collaboration, such as not sharing the same physical space, created a barrier, making it harder to mobilize these resources. Residents visually represented this challenge by placing unfamiliar professionals in distant locations on their sociograms. Unfamiliar professionals were most frequently positioned in the outer concentric circle, and consisted of supervisors (Figure 3, 45%), and program directors (Figure 3, 12%). For example, the lack of direct interaction meant that residents sometimes had to consult a supervisor they knew only through phone conversations, making effective communication more challenging (Resident 26).

To cope with this unfamiliarity, residents sought guidance from more accessible network members, like nurses, approachable supervisors, or peers, especially when they felt uncomfortable reaching out to an unfamiliar supervisor. One resident expressed feeling closer to a nurse than to her supervisor due to limited in-person interactions with supervisors, highlighting the importance of comfort and support in navigating their networks. This aligns with Figures 3 and 4, which show that nurses were the most frequently identified alters (25% negative situations, 24% positive situations) in the closest concentric circle.

Beyond physical proximity, prior relationships also influenced help-seeking. Residents found it easier to approach supervisors, senior specialty residents, or nurses with whom they had worked previously. As a result, these individuals were often positioned close to one another in the sociograms, indicating their perceived accessibility for expertise related to patient care goals. One resident explained how a dual role strengthened their connection:

*[Name supervisor] is also my research supervisor, so I contact him often. He is younger.… I can ask him [more easily]; I find him more approachable than [name supervisor 2]. (Resident 23)*

### 3.2 (Un)Awareness of the Supportive Potential

Residents struggled to understand the potential that other healthcare professionals could bring to collaborative efforts, particularly in complex patient cases that require input from multiple specialties. Determining each professional’s expertise was challenging, especially for inexperienced residents in emergency departments. In one case, a resident sought advice from specialists, each focused only on their subspecialty, making it difficult to maintain oversight of the patient’s case. Without a clear understanding of whom to turn to for broader guidance, she struggled to communicate her need for help, leading to frustration and a sense of being overwhelmed:

*At first, it seemed to be possible [to solve the patient’s case]. And then it became more difficult, and after a long time, I understood: I cannot do it anymore. Although I said it [i.e., I cannot handle it anymore], I think I was not clear enough. (Resident 27)*

The resident’s difficulty in expressing her need for help could indicate that she was uncertain about the supervisor’s specific expertise in navigating complex cases or that she was overwhelmed and could not communicate her need for help clearly.

**Figure 2:**
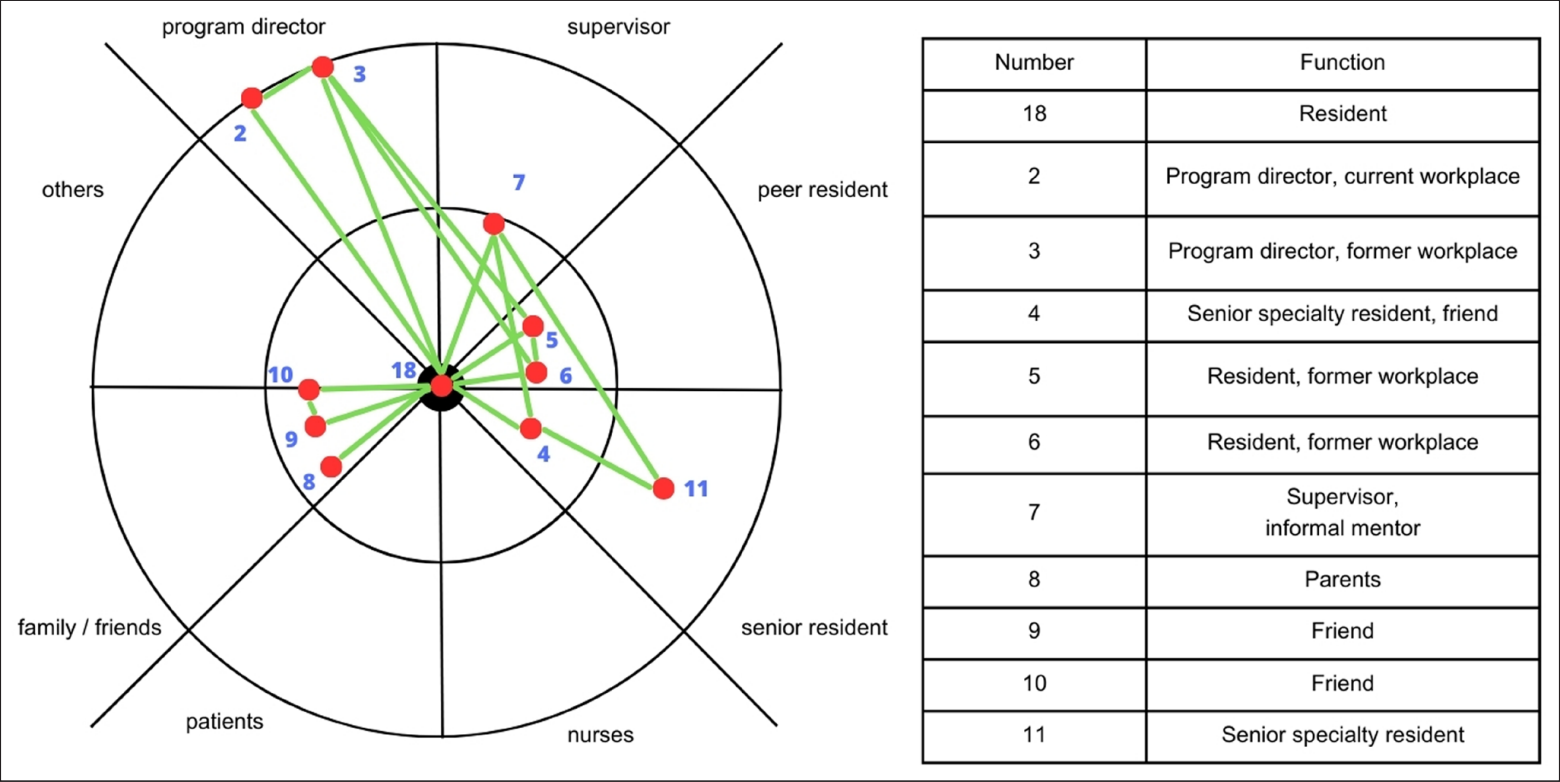
Resident 18’s sociogram, showing which connections are closely connected, and which are not. *Note:* This figure illustrates the ego network of Resident 18 in a ‘negative situation’ – being declined for a specialty training position. Resident 18 is centrally located in the sociogram, with 11 alters from five role groups: supervisors, peer residents, senior residents, family/friends, and program directors. In this scenario, Resident 18 perceives the Program Directors (2,3) as inaccessible, placing them in the outer concentric circle and avoiding direct clarification about her application. Instead, she seeks advice from peer residents (5,6), senior residents (4,11), parents (8), and friends (9,10) – all positioned in the closest (4,5,6,8,9,10) and middle (11) concentric circles, indicating greater perceived accessibility. Supervisor (7) assists with CV improvement but does not take on a mentoring role in the application process. Positioned in the middle concentric circle, the supervisor is somewhat accessible but not a primary source of career guidance.

**Figure 4:**
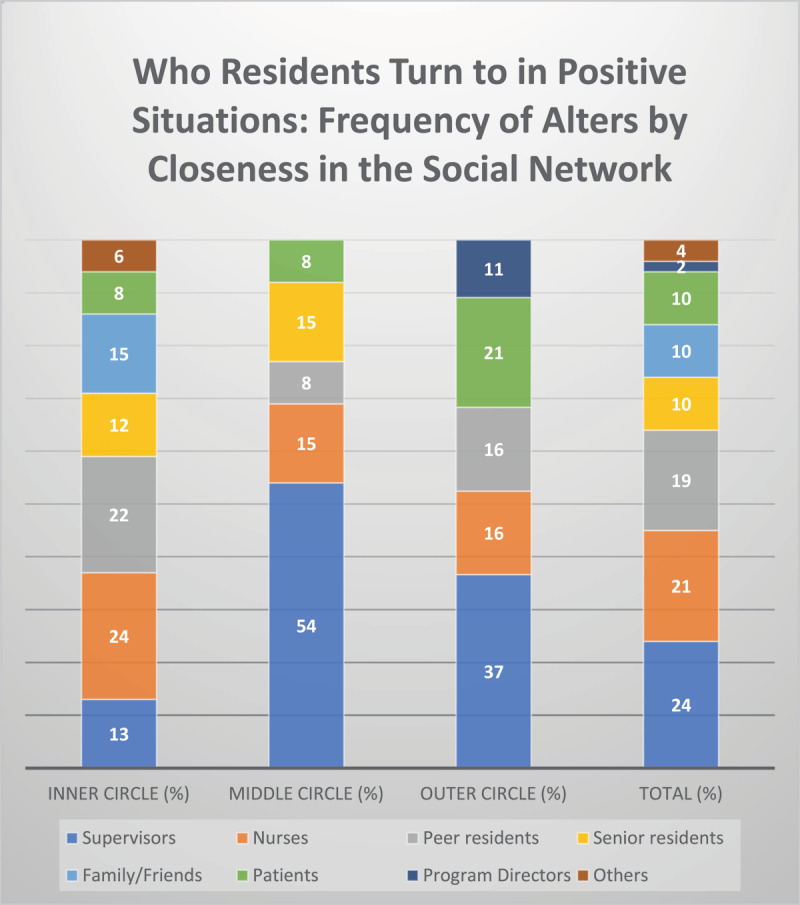
Visual representation of Residents’ Social Capital Mobilization in Positive Situations. This figure presents an overview of residents’ social capital mobilization networks in positive situations. It shows which groups of alters residents placed in the inner, middle, and outer circles. The final bar displays the overall distribution of these groups across all circles.

Similar uncertainties shaped how residents sought career-related guidance. Resident 18 (see Figure 2) encountered psychological barriers when seeking advice about specialty training applications. The resident found it difficult to ask a program director for clarification on how to strengthen her CV and was unsure whether to approach a supervisor to discuss her doubts about continuing in her specialty. Instead, she relied on peers, family, and friends for support, even though they lacked the professional insight that might have been helpful. This pattern was not unique: as shown in Figures 3 and 4, family and friends were frequently identified as alters in both negative (10%) and positive (10%) situations. While a supervisor in her sociogram helped refine her CV, the interaction remained surface-level and did not extend into a deeper conversation about her motivations or career goals. This may suggest a limited understanding of the supervisor’s potential value as a mentor, and perhaps a mutual lack of awareness about how they could support one another. It might also be that approaching the supervisor is less desirable because the resident might not feel comfortable starting the conversation. Figure 2 illustrates how perceptions of the inaccessibility of program directors and the limited mentoring role of supervisors influenced her help-seeking behavior.

In both cases, the residents’ struggles reflect the broader issue of unawareness of the supportive potential of their professional networks. The missed opportunities to involve supervisors or program directors demonstrate a need for clearer communication strategies, as well as a better understanding of the values of professionals in their network.

### 3.3 Difficulty accessing the right person

Timely access to the right persons was another significant challenge. Residents found it difficult to communicate the urgency of situations effectively to supervisors, often leading to misunderstandings and inadequate support. As one resident noted:

*While the patient needed critical care … I did not ask explicitly: ‘Can you come’, but instead: ‘The patient is very bad, and I am not on call anymore’. (Resident 22)*

This example highlights residents’ ongoing development in professional communication and network utilization. They are still learning how to navigate the system effectively, particularly when it comes to making explicit requests for assistance. For instance, residents often encountered mismatches between the type of supervision they expected and what they received. Their requests for a supervisor’s physical presence were frequently implicit rather than direct, resulting in remote supervision via telephone, which limited the exchange of expertise. As a consequence, residents felt unsupported because there were misalignments between the type of support they expected and what they received. The data suggests that residents, particularly in critical situations, do not always recognize or engage the most valuable members of their network. As seen in Table 2, in negative situations, residents tend to have more alters positioned in the outer circle, whereas in positive situations, alters are more concentrated in the inner circle. This indicates that in challenging situations, residents may struggle to identify and mobilize the most relevant resources, reinforcing the importance of understanding their network’s value.

To overcome the challenge of accessing other healthcare professionals in a timely manner, residents explored understanding the expertise of various professionals, such as nurses. Acknowledging the unique resources that network members bring, fosters collaboration and enhances patient care. As one resident noted:

*It is good to acknowledge: what does the other [i.e., nurse] know? And, the nurses do know other things where I do not have any expertise. (Resident 34)*

By leveraging the strengths of various professionals within their network, residents can enhance their ability to navigate challenges effectively.

### 3.4 Perceived high costs of seeking support

Residents often perceived seeking resources from certain others, such as certain supervisors and program directors, as too costly. The perceived inaccessibility of program directors is further supported by data from Figures 3, 4, and Table 2, which show that, in both negative and positive situations, PDs are never placed in the closest concentric circle. Additionally, supervisors are the alters most frequently identified in the outer circle, appearing in 45% (n = 15) of negative situations and 37% (n = 7) of positive situations, further emphasizing their perceived distance and limited accessibility. These structural barriers contributed to residents’ fear of asking for resources, as they worried that doing so could jeopardize their chances of obtaining a specialty training position, creating a psychological barrier. This fear was exacerbated by concerns about being perceived negatively or being judged for seeking help, making them hesitant to approach these individuals. In some cases, residents experienced psychologically unsafe behaviors, such as supervisors gossiping about residents’ performance, further reinforcing their reluctance to seek support:

*You should not comment on someone’s performance when they’re not there, especially not a supervisor, … it creates an unsafe environment. (Resident 27)*

Female residents faced additional challenges, such as being questioned about their ability to balance motherhood with a career as a specialty resident. These gendered expectations contributed to psychological barriers, making it harder to speak out against inappropriate behavior.

To overcome these psychological barriers, residents employed various strategies to manage the perceived cost of seeking help. These included delaying requests for expertise until absolutely necessary, bundling multiple issues into a single request to reduce the frequency of seeking help, or seeking help from more approachable network members, such as senior residents and nurses, who posed a lower risk of professional repercussions. By strategically selecting whom to approach, residents weighed the risks and benefits of seeking help from different figures and adjusted their strategies accordingly. By seeking alternative resources within their network, they managed to overcome barriers while minimizing potential costs to their professional trajectory.

## 4. Discussion

The study explored how residents perceive and respond to barriers in accessing and making use of (or mobilizing) social capital during challenging situations. Residents identified a range of physical and psychological barriers to accessing and making use of social capital, which were illustrated through ego network sociograms that mapped their interactions. Key barriers included not knowing certain colleagues, which led residents to rely more on accessible figures, such as familiar supervisors or nurses. Uncertainty about the expertise of various professionals also caused hesitation, resulting in missed opportunities for guidance. Additionally, residents communicated implicitly rather than making explicit requests for help, contributing to feelings of inadequate support during critical moments. Seeking help from authoritative figures was perceived as socially costly, deterring timely supervision. These challenges have serious implications for patient care quality [43,44], as hesitation to seek timely guidance or recognize expertise can lead residents to handle situations beyond their competence, increasing the risk of errors [45,46]. Supportive and accessible supervision plays a vital role in mitigating these risks. However, inconsistent or unsafe supervision can leave residents feeling unsupported, delaying critical decisions, and potentially resulting in suboptimal patient outcomes. These findings resonate with previous research showing that residents face challenges in seeking expertise from supervisors who hold evaluative power and influence over access to specialty training positions [3,11,47].

Our findings indicate that navigating social networks to obtain necessary resources is complex and fraught with challenges, particularly due to the power dynamics present in the hierarchical learning environment in clinical settings [3,4,8]. While residents may find it easier to seek resources from senior residents or nurses due to established rapport [3,4,47], the decision to seek help is heavily influenced by hierarchical relationships. The perceived ‘costs’ of seeking help from supervisors can be explained by power differentials and concerns about the judgment of professional repercussions. Supervisors who engage in gossip or other informal power structures may exacerbate these concerns. Gossip, in particular, is often used to reinforce group norms and typically targets lower-status individuals, like residents [48]. The aspects of gossiping and informal power structures may influence not only the residents’ current access to resources but also their long-term professional trajectory, particularly in relation to specialty training positions.

Interestingly, while hierarchy typically suggests a clear structure of authority [8], our sociograms indicated that not all supervisors were perceived as distant; those supervisors whom residents already knew from previous jobs or roles were often perceived as more accessible. This highlights the nuance in how residents navigate their social networks – accessibility is not simply determined by formal hierarchy but by dynamics between supervisors and residents and experiences from previous interactions [15]. As seen in the results (Figures 3, 4, and Table 2), supervisors were often placed in both the closest and outermost circles of the network, reflecting the variability in perceived accessibility based on prior interactions and the social dynamics of the environment.

Our findings suggest that power dynamics are a critical yet underrepresented factor in current models of help-seeking, including Borgatti and Cross’s model, which tends to downplay the importance of power in information and advice-seeking behavior [15]. While the model effectively addresses general costs related to information-seeking, it does not fully capture the weight of power-related barriers. In our study, power and fear of judgment were prominent barriers to seeking help [3], leading us to propose that the model be revised to include power dynamics as a determinant in help-seeking behavior explicitly. A revised model could be created by conceptualizing power as an integral component of the ‘cost’ of seeking help rather than treating it as a background factor. The revised model would reflect that in environments with clear hierarchical structures, power can significantly influence the decision to seek help, with residents weighing not only the cost of seeking help, but also the potential professional risks associated with approaching higher-status individuals.

**Figure 5:**
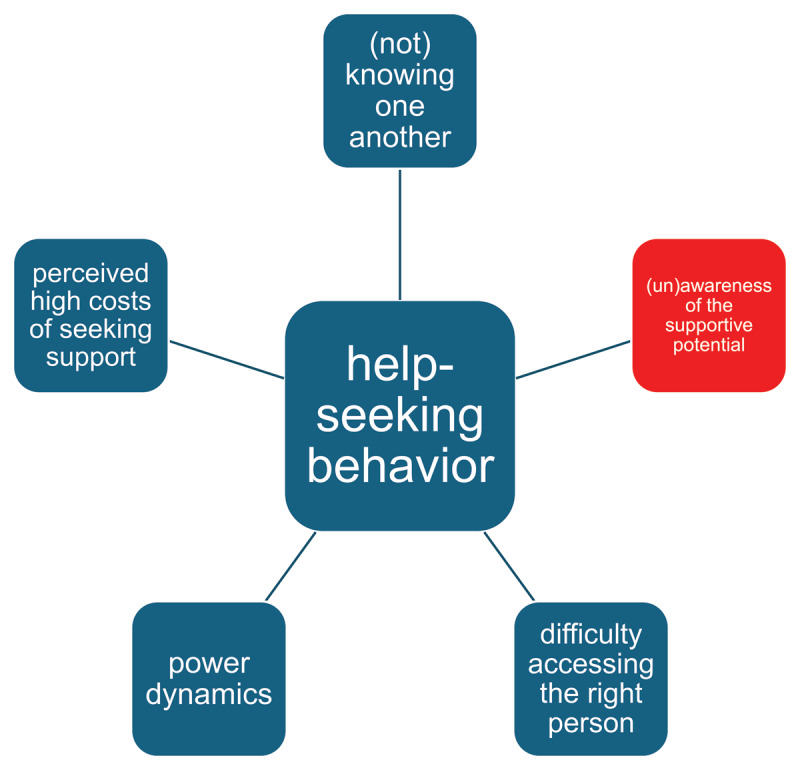
Antecedents of Help-Seeking (challenges) in Residency (derived from Borgatti & Cross, 2003).

In addition, we found that the original model’s focus on “knowing someone’s value” did not align closely with how residents described their decision-making. Rather than lacking information about what value a person could offer, residents often expressed uncertainty about how to approach others or whether it was appropriate to do so. We therefore rephrase this element of the model as “(Un)Awareness of the Supportive Potential”, which better captures how help-seeking is shaped by role clarity, perceived accessibility, and relational comfort. This element may be further influenced by the focus of residents’ training that emphasizes autonomy, leaving residents less attuned to the potential supportive roles others can play in clinical settings [3,11]. The revised model is schematically represented in Figure 5 (Appendix).

### 4.1. Practical Implications

Our findings suggest that residents should be encouraged to build a broad network of social relationships to navigate workplace challenges better. This can be supported by workshops or training focused on reflecting on accessing and making use of social capital, identifying barriers, and developing strategies to overcome them. Reflective discussions aimed at improving communication between residents and healthcare team members are essential [49].

For supervisors, the results highlight that while they may perceive themselves as approachable, residents may not always share this perception, potentially leading them to seek support elsewhere. Proactive measures, such as clarifying their availability to junior residents, providing bedside information, and involving senior residents and nurses in supervision, can help bridge this gap. These insights can inform faculty development programs, emphasizing the importance of fostering social relationships and promoting effective collaboration within hierarchical structures.

### 4.2. Limitations and Further Research

A limitation of this study is that the first phase involved secondary data analysis of pre-existing data, which was not conceptualized originally from a social capital perspective. Instead, the primary focus of this initial analysis was on residents’ adaptation strategies during their first year and the support they received [37]. To address the potential limitation of superficial insights from data not explicitly designed to explore social capital, these findings were systematically used to develop and refine the interview guide for the subsequent data collection, thereby enhancing the depth and relevance of the insights generated [24]. Despite the richness of the data, this cross-sectional qualitative study cannot infer causality. We sampled residents at two early career stages: residents not in training and second-year residents reflecting on their first year of residency. While providing valuable insights, these responses do not reveal how residents’ ability to mobilize social capital evolves throughout their training. Future longitudinal research could investigate changes in residents’ social networks and social capital during their training [26,50]. Since transitions are often stressful [51], studying how network changes affect well-being and performance during training would be valuable.

Another limitation is that each resident identified their work-related social network in a specific situation rather than their entire social network at that moment, which offers a limited view of how they accessed and made use of social capital in other contexts. However, using sociograms during interviews enhanced recall of their memories of the social actors in their networks by providing structure [27]. Sociograms revealed that the hierarchy level did not necessarily correlate with a supervisor’s approachability. They also clarified the meanings of distance and closeness: distance could indicate power dynamics or unfamiliarity, while closeness highlighted approachability. Thus, sociograms helped uncover how residents leveraged close relationships to overcome barriers [26,28].

While our study focuses on a Western healthcare setting, we acknowledge that residents may mobilize social capital differently in non-Western contexts due to variations in healthcare systems, hierarchies, and cultural norms. We did not collect data on residents’ social backgrounds, which could influence these dynamics. However, prior research indicates that residents in the Netherlands often come from higher socioeconomic, non-immigrant backgrounds, potentially easing access to support [52]. Future research should examine how background and cultural factors shape these processes in diverse contexts.

Finally, future research should also further assess whether the proposed addition of power dynamics and “(un)awareness of the supportive potential” to the Borgatti and Cross model is transferable to other groups.

### 4.3. Conclusion

Our study reveals how residents perceive and respond to barriers in accessing and making use of social capital during challenging situations. Unfamiliarities with colleagues, such as knowing a supervisor only through phone conversations, limit trust and support. Uncertainty about available resources, like which specialist to consult for specific expertise or who to contact to explore specialty training opportunities. Residents also perceive seeking support as socially costly, fearing judgment or criticism for asking for help. This is worsened by the hierarchical structure of healthcare, where supervisors and program directors hold evaluative power over residents’ career trajectories. To cope, residents rely on accessible colleagues like senior residents and nurses, emphasizing the role of familiarity in accessing expertise. They also turn to friends and family for support when professional guidance feels out of reach. These findings highlight the complexity of residents’ social networks and the need to foster supportive relationships within healthcare teams.

Our results further suggest that existing models of help-seeking, such as Borgatti and Cross’s, may need refinement to better reflect the realities of clinical environments. We propose two key adaptations: first, power dynamics should be conceptualized as an integral part of the ‘cost’ of help-seeking within a hierarchical system, given the fear of judgment in residents’ decision-making. Second, rather than focusing on “knowing someone’s value,” the model should reflect the uncertainty residents experience about *awareness of the supportive potential* — acknowledging that help-seeking is shaped not only by information gaps but also by relational dynamics, role ambiguity, and social comfort.

Creating a psychologically safe environment can encourage help-seeking, enhance workplace navigation, and improve patient care. Faculty development programs should prioritize collaborative networks and promote effective communication strategies.

## Data Accessibility Statement

The participants of this study did not give written consent for their data to be shared publicly, so due to the sensitive nature of the research supporting data is not available.

## Appendix 2

**Interview guide 1**

The exploratory, in-depth interviews were conducted around the central question: ‘How did you experience your first year as a resident?’.

*Introductory Interview*

– Where did you study, and what was your experience after graduation?
– What was your role and what were your responsibilities?
– How long have you been in your current position as a resident in training?
– In which departments have you worked and what were the differences?
– What were your responsibilities during your first year as a resident?

*Core Interview Phase*

– How did you experience your first year as a resident in training?
– Did you feel pressure, and if so, under what circumstances?
– Did you experience stress, and if so, what were the reasons for the stress?
– How do you reflect on the expected/unexpected responsibilities of your first year?
– Why did you find some responsibilities unexpected?
– Did you experience support from your colleagues? From whom? And where does this support come from?
– Did you receive feedback? From whom and how did you perceive this feedback?
– Did you feel valued in your work? By whom and for what reasons?
– Who have you learned a lot from? And for what purposes?

## Appendix 3

**Interview guide 2**

Background information

– What is your gender?
– What is your age?
– In what specialty do you work?
– How many months have you worked since graduating from medical school?
– Do you already work in ‘on-call situations’?

The interview consists of 4 parts:

Part 1: If you need any help in your work as a resident, who do you ask, for what, and why (for what goal)?

Part 2: Can you describe a challenging situation in which you needed help, but you were unable to get this help, or you had the feeling of being lonely?

– What happened in this situation?
– Who was present in this situation?
– Who was absent in this situation, while you would like that they would be there?
– Can you write down all the people involved in the situation on Post-its
– Can you place the Post-it in one of the categories, and within one of the circles? If you place people closer, it means that you easily approach that specific person.
– What did you miss from the absent person and why? What support did you want to receive?
– What was the consequence of the lack of support (did you receive your goal yes or not)
– What is the relation between people on the sociogram, can you draw a line if they know each other?

Part 3: Can you describe a challenging situation in which you needed help and you got the help you wanted?

– What happened in this situation?
– Who was present?
– Can you write down all the people involved in the situation on Post-its
– Can you place the Post-it in one of the categories, and within one of the circles? If you place people closer, it means that you easily approach that specific person.
– From whom did you receive help and for what goal?
– Why were you able to get help in this situation?
– What is the relation between people on the sociogram, can you draw a line if they know each other?

Part 4: If you compare sociograms of both situations. Why were you in the first situation unable to get help, why were you able to get help in the second situation? Can you reflect on that?

## Appendix 4

**Table 2:** Comparison of Social Network Mobilization in Negative and Positive Situations.

CHARACTERISTIC	NEGATIVE SITUATION (n = 18)	POSITIVE SITUATION (n = 13)

Mean network size (alter count) (smallest – largest)	7 (3–15)	8 (3–12)

Total alters	124	99

Mean alters per circle		

Inner	4 (65/18)	5 (67/13)

Middle	1 (26/18)	1 (13/13)

Outer	2 (33/18)	1 (19/13)

Most frequently identified alters (from most to least frequent):	Supervisors (n = 43, 35%), nurses (n = 19, 15%), peer resident (n = 13, 10%), family/friends (n = 13, 10%) senior resident (n = 13, 10%), patients (n = 9, 7%), other (n = 8, 6%), program director (n = 6, 5%)	Supervisors (n = 23, 23%), nurses (n = 21, 21%), peer resident (n = 19, 19%), senior resident (n = 10, 10%), family/friends (n = 10, 10%), patients (n = 10, 10%), other (n = 4, 4%), program director (n = 2, 2%)

Most frequently identified alters in the closest concentric circle (from most to least frequent) (% = number of alters/total number of alters per circle):	Nurses (n = 16, 25%), family/friends (n = 13, 22%, supervisors (n = 11, 17%), peer residents (n = 9, 14%), senior resident (n = 8, 12%), others (n = 5, 8%), patients (n = 3, 5%), program director (n = 0)	Nurses (n = 16, 24%), peer residents (n = 15, 22%), family/friends (n = 10, 15%), supervisors (n = 9, 13%), senior resident (n = 8, 12%), patients (n = 5, 8%), others (n = 4, 6%), program director (n = 0)

Most frequently identified alters in the middle concentric circle (from most to least frequent) (% = number of alters/total number of alters per circle):	Supervisor (n = 17, 65%), peer residents (n = 3, 12%), senior residents (n = 3, 12%), program directors (n = 2, 8%), nurses (n = 1, 4%), patients (n = 0), family/friends (n = 0), other (n = 0)	Supervisors (n = 7, 54%) senior residents (n = 2, 15%), nurses (n = 2, 15%), peer resident (n = 1, 8%) patient (n = 1, 8%), family/friends (n = 0), program director (n = 0), other (n = 0)

Most frequently identified alters in the outer concentric circle(from most to least frequent) (% = number of alters/total number of alters per circle):	Supervisors (n = 15, 45%), patients (n = 6, 18%), program director (n = 4, 12%), others (n = 3, 9%), senior resident (n = 2, 6%), nurses (n = 2, 6%), peer resident (n = 1, 3%), family/friends (n = 0)	Supervisors (n = 7, 37%), patients (n = 4, 21%), peer resident (n = 3, 16%), nurses (n = 3, 16%), program director (n = 2, 11%), senior resident (n = 0), family/friends (n = 0), others (n = 0)

This table provides an overview of the residents’ social networks in both negative and positive situations. It illustrates differences in network size, the distribution of alters across concentric circles, and the most frequently identified alters in each scenario. The table highlights how residents access and make use of social capital differently depending on the context, with notable variations in accessibility and support structures.

## Abbreviations

CV: curriculum vitae, SNA: social network analysis, AN: Aly Najak, DJ: Debbie Jaarsma, GG: Gerbrich Galema, GW: Götz Wietasch, JB: Jasperina Brouwer, PD: program director, TA: Tobi Ajayi, WL: Wally van der Laan, YtH: Yvonne ten Hoeve

## References

[1] Dyrbye L, Shanafelt T. A narrative review on burnout experienced by medical students and residents. Med Educ. 2016 Jan 1;50(1):132–49. DOI: 10.1111/medu.12927 26695473

[2] Kilminster S, Cottrell D, Grant J, Jolly B. AMEE Guide No. 27: Effective educational and clinical supervision. Medical Teacher. 2007;29:2–19. DOI: 10.1080/01421590701210907 17538823

[3] Jansen I, Stalmeijer RE, Silkens MEWM, Lombarts KMJMH. An act of performance: Exploring residents’ decision-making processes to seek help. Med Educ. 2021;55(6):758–67. DOI: 10.1111/medu.14465 33539615 PMC8247982

[4] Jansen I, Silkens MEWM, Galema G, Vermeulen H, Geerlings SE, Lombarts KMJMH, et al. Exploring nurses’ role in guiding residents’ workplace learning: A mixed-method study. Med Educ. 2022;(October):1–12. DOI: 10.1111/medu.14951 36226355

[5] Olmos-Vega FM, Dolmans DHJM, Guzmán-Quintero C, Echeverri-Rodriguez C, Teunnissen PW, Stalmeijer RE. Disentangling residents’ engagement with communities of clinical practice in the workplace. Advances in Health Sciences Education. 2019;24(3):459–75. DOI: 10.1007/s10459-019-09874-9 30659426

[6] Bannister SL, Dolson MS, Lingard L, Keegan DA. Not just trust: factors influencing learners’ attempts to perform technical skills on real patients. Med Educ. 2018;52(6):605–19. DOI: 10.1111/medu.13522 29446155

[7] Teunissen PW, Watling CJ, Schrewe B, Asgarova S, Ellaway R, Myers K, et al. Contextual Competence: How residents develop competent performance in new settings. Med Educ. 2021;(February):1–10. DOI: 10.1111/medu.14517 PMC8451833 33630305

[8] Witman Y. What do we transfer in case discussions? The hidden curriculum in medicine…. Perspect Med Educ. 2014 Apr 1;3(2):113–23. DOI: 10.1007/s40037-013-0101-0 24366760 PMC3976482

[9] MacNeil KA, Regehr G, Holmes CL. Contributing to the hidden curriculum: exploring the role of residents and newly graduated physicians. Advances in Health Sciences Education. 2022 Mar 1;27(1):201–13. DOI: 10.1007/s10459-021-10081-8 34822055

[10] Wiese A, Kilty C, Bennett D. Supervised workplace learning in postgraduate training: a realist synthesis. Vol. 52, Medical Education. 2018;52:951–69. Blackwell Publishing Ltd. DOI: 10.1111/medu.13655

[11] Kennedy TJT, Regehr G, Baker GR, Lingard L. Preserving professional credibility: Grounded theory study of medical trainees’ requests for clinical support. BMJ (Online). 2009;338(7691):399–401. DOI: 10.1136/bmj.b128 PMC2640114 19204035

[12] Bernabeo EC, Holtman MC, Ginsburg S, Rosenbaum JR, Holmboe ES. Lost in transition: The experience and impact of frequent changes in the inpatient learning environment. Academic Medicine. 2011;86(5):591–8. DOI: 10.1097/ACM.0b013e318212c2c9 21436668

[13] Christakis DA, Feudtner C. Temporary matters: The ethical consequences of transient social relationships in medical training. JAMA. 1997;278(9):739–43. DOI: 10.1001/jama.278.9.739 9286834

[14] Englander R, Carraccio C. A Lack of Continuity in Education, Training, and Practice Violates the “Do No Harm” Principle. Academic Medicine. 2018;93(3 S):S12–6. DOI: 10.1097/ACM.0000000000002071 29485481

[15] Borgatti SP, Cross R. A relational view of information seeking and learning in social networks. Manage Sci. 2003;49(4):432–45. DOI: 10.1287/mnsc.49.4.432.14428

[16] Law M, Lam M, Wu D, Veinot P, Mylopoulos M. Changes in personal relationships during residency and their effects on resident wellness: A qualitative study. Academic Medicine. 2017;92(11):1601–6. DOI: 10.1097/ACM.0000000000001711 28445221 PMC5662155

[17] Lin N. Social networks and status attainment. Annu Rev Sociol. 1999;25(Weber 1946):467–87. DOI: 10.1146/annurev.soc.25.1.467

[18] Coleman JS. Social Capital in the Creation of Human Capital. American Journal of Sociology (Supplement). 1988;94:S95–S120. DOI: 10.1086/228943

[19] Wasserman S, Faust K. Social Network Analysis in the Social and Behavioral Sciences. Social Network Analysis. 1994;3–27. 10.1017/CBO9780511815478.002

[20] Kadushin C. Basic Network Concepts, Part I. Understanding Social Networks; 2012. pp. 13–26.

[21] Lin N. Social Capital, a theory of social structure and action. Cambridge: Cambridge University Press; 2001. pp. 41–54. DOI: 10.1017/CBO9780511815447

[22] Borgatti SP. Analyzing social networks. Los Angelos: SAGE Publications; 2018.

[23] Bunniss S, Kelly DR. Research paradigms in medical education research. Med Educ. 2010;44(4):358–66. DOI: 10.1111/j.1365-2923.2009.03611.x 20444071

[24] Heaton J. Reworking Qualitative Data [Internet]. London: SAGE Publications Ltd; 2004. Available from: https://methods.sagepub.com/book/reworking-qualitative-data DOI: 10.4135/9781849209878

[25] Malterud K, Siersma VD, Guassora AD. Sample Size in Qualitative Interview Studies: Guided by Information Power. Qual Health Res [Internet]. 2016;26(13):1753–60. DOI: 10.1177/1049732315617444 26613970

[26] Froehlich DE, Brouwer J. Social network analysis as mixed analysis. In: The Routledge Reviewer’s Guide to Mixed Methods Analysis. Taylor and Francis; 2021. pp. 209–17. DOI: 10.4324/9780203729434-19

[27] McCarty C, Luis Molina J, Aguilar C, Rota L. A comparison of social network mapping and personal network visualization. Field methods. 2007;19(2):145–62. DOI: 10.1177/1525822X06298592

[28] Nimmon L, Atherley A. Qualitative ego networks in health professions education: Capturing the self in relation to others. Med Educ. 2022;56(1):71–81. DOI: 10.1111/medu.14663 34490649

[29] Laengler M, Brouwer J, Gruber H. Data collection for mixed method approaches in social network analysis. In: Froehlich DE, Rehm M, Rienties BC, editors. Mixed Methods Social Network Analysis. 1st ed. Routledge; 2019. DOI: 10.4324/9780429056826-4

[30] Van Waes S, Van den Bossche P, Moolenaar NM, De Maeyer S, Van Petegem P. Know-who? Linking faculty’s networks to stages of instructional development. High Educ (Dordr). 2015;70(5):807–26. DOI: 10.1007/s10734-015-9868-8

[31] Atherley AEN, Nimmon L, Teunissen PW, Dolmans D, Hegazi I, Hu W. Students’ social networks are diverse, dynamic and deliberate when transitioning to clinical training. Med Educ [Internet]. 2021 Mar 13;55(3):376–86. DOI: 10.1111/medu.14382 32955741 PMC7984257

[32] Weggemans MM, van Dijk B, van Dooijeweert B, Veenendaal AG, ten Cate O. The postgraduate medical education pathway: An international comparison. GMS J Med Educ. 2017;34(5):1–16. DOI: 10.3205/zma001140 PMC5704606 29226231

[33] Van der Velde F, Van de Leemkolk B, Lodder A. Loopbanen en loopbaanwensen van basisartsen. Meting 2019. 2019;(oktober):139.

[34] Ten Cate O, Taylor DR. The recommended description of an entrustable professional activity: AMEE Guide No. 140. Med Teach. 2021;43(10):1106–14. DOI: 10.1080/0142159X.2020.1838465 33167763

[35] Dijkhuizen K, Bustraan J, van den Bogaard MED, Velthuis SI, van Lith JMM, Driessen EW, et al. Values and beliefs on trainee selection: What counts in the eye of the selector? A qualitative study exploring the program director’s perspective. Med Teach [Internet]. 2020;42(10):1179–86. DOI: 10.1080/0142159X.2020.1798912 32755426

[36] Het College Geneeskundige Specialismen. Kaderbesluit Geneeskundig Specialisme;. 2019. pp. 1–87. Available from: https://www.knmg.nl/opleiding-herregistratie-carriere/cgs/regelgeving/specialismen.htm

[37] Galema G, Brouwer J, Bouwkamp-Timmer T, Jaarsma DADC, Wietasch GJKG, Duvivier RRJ. Transitioning to residency: a qualitative study exploring residents’ perspectives on strategies for adapting to residency. BMC Med Educ. 2025 Jan 2;25(1):6. DOI: 10.1186/s12909-024-06565-x 39748348 PMC11697482

[38] Varpio L, Ajjawi R, Monrouxe LV, O’Brien BC, Rees CE. Shedding the cobra effect: Problematising thematic emergence, triangulation, saturation and member checking. Med Educ. 2017;51(1):40–50. DOI: 10.1111/medu.13124 27981658

[39] ATLAS.ti Scientific Software Development GmbH B. ATLAS.ti [Internet]. Available from: https://atlasti.com/

[40] Irwin S. Qualitative secondary data analysis: Ethics, epistemology and context. Progress in Development Studies. 2013;13(4):295–306. DOI: 10.1177/1464993413490479

[41] Ramani S, Könings KD, Mann K, van der Vleuten CPM. A Guide to Reflexivity for Qualitative Researchers in Education. Acad Med. 2018 Aug;93(8):1257. DOI: 10.1097/ACM.0000000000002263. 29697429

[42] Finlay L, Gough B. Reflexivity: A Practical Guide for Researchers in Health and Social Sciences. Blackwell Science; 2003. DOI: 10.1002/9780470776094

[43] Farnan JM, Petty LA, Georgitis E, Martin S, Chiu E, Prochaska M, Arora VM. A systematic review: the effect of clinical supervision on patient and residency education outcomes. Acad Med. 2012 Apr;87(4):428–42. DOI: 10.1097/ACM.0b013e31824822cc 22361801

[44] Jagsi R, Kitch BT, Weinstein DF, Campbell EG, Hutter M, Weissman JS. Residents Report on Adverse Events and Their Causes. Arch Intern Med. 2005;165(22):2607–2613. DOI: 10.1001/archinte.165.22.2607 16344418

[45] Rothwell C, Kehoe A, Farook SF, Illing J. Enablers and barriers to effective clinical supervision in the workplace: A rapid evidence review. BMJ Open. 11. DOI: 10.1136/bmjopen-2021-052929 PMC8479981 34588261

[46] Derse AR. Medical Training and Errors: Competence, Culture, Caring, and Character. Acad Med. 2020 Aug;95(8):1155–1158. DOI: 10.1097/ACM.0000000000003118 31833851

[47] Eppich WJ, Dornan T, Rethans JJ, Teunissen PW. “Learning the Lingo”: A Grounded Theory Study of Telephone Talk in Clinical Education. Acad Med. 2019 Jul;94(7):1033–1039. DOI: 10.1097/ACM.0000000000002713 30893065

[48] Ellwardt L, Labianca GJ, Wittek R. Who are the objects of positive and negative gossip at work? A social network perspective on workplace gossip. Soc Networks. 2012 May;34(2):193–205. DOI: 10.1016/j.socnet.2011.11.003

[49] Sklar DP. Leadership in Academic Medicine: Purpose, People, and Programs. Acad Med. 2018 Feb;93(2):145–148. DOI: 10.1097/ACM.0000000000002048 29377851

[50] Audulv Å, Hall EOC, Kneck Å, Westergren T, Fegran L, Pedersen MK, et al. Qualitative longitudinal research in health research: a method study. BMC Med Res Methodol. 2022 Dec 1;22(1). DOI: 10.1186/s12874-022-01732-4 PMC9526289 36182899

[51] Teunissen PW, Westerman M. Opportunity or threat: The ambiguity of the consequences of transitions in medical education. Med Educ. 2011;45(1):51–9. DOI: 10.1111/j.1365-2923.2010.03755.x 21155868

[52] Mulder L, Wouters A, Akwiwu EU, Koster AS, Ravesloot JH, Peerdeman SM, Salih M, Croiset G, Kusurkar RA. Diversity in the pathway from medical student to specialist in the Netherlands: a retrospective cohort study. Lancet Reg Health Eur. 2023 Oct 12;35:100749. DOI: 10.1016/j.lanepe.2023.100749 37860636 PMC10583163

